# Randomized controlled trial to evaluate tooth stain reduction with nicotine replacement gum during a smoking cessation program

**DOI:** 10.1186/1472-6831-12-13

**Published:** 2012-06-13

**Authors:** Helen Whelton, Rose Kingston, Denis O’Mullane, Frederick Nilsson

**Affiliations:** 1 Oral Health Services Research Centre, University College Cork, Ireland; 2 Global Medical Affairs & Clinical Research, McNeil AB, Norrbroplatsen 2, SE-25109, Helsingborg, Sweden

## Abstract

**Background:**

In addition to its general and periodontal health effects smoking causes tooth staining. Smoking cessation support interventions with an added stain removal or tooth whitening effect may increase motivation to quit smoking. Oral health professionals are well placed to provide smoking cessation advice and support to patients. The objective of the present study was to evaluate the effect of Nicorette® Freshmint Gum used in a smoking cessation programme administered in a dental setting, on extrinsic stain and tooth shade among smokers.

**Methods:**

An evaluator-blinded, randomized, 12-week parallel-group controlled trial was conducted among 200 daily smokers motivated to quit smoking. Participants were randomised to use either the Nicorette® Freshmint Gum or Nicorette® Microtab (tablet). Tooth staining and shade were rated using the modified Lobene Stain Index and the Vita® Shade Guide at baseline, weeks 2, 6 and 12. To maintain consistency with other whitening studies, the primary end-point was the mean change in stain index between baseline and week 6. Secondary variables included changes in stain measurements and tooth shade at the other time points the number of gums or tablets used per day and throughout the trial period; and the number of cigarettes smoked per day. Treatments were compared using analysis of covariance (ANCOVA), using treatment and nicotine dependence as factors and the corresponding baseline measurement as a covariate. Each comparison (modified intention-to-treat) was tested at the 0.05 level, two-sided. Within-treatment changes from baseline were compared using a paired *t*-test.

**Results:**

At week 6, the gum-group experienced a reduction in mean stain scores whilst the tablet-group experienced an increase with mean changes of -0.14 and +0.12 respectively, (p = 0.005, ANCOVA). The change in mean tooth shade scores was statistically significantly greater in the gum-group than in the tablet group at 2 (p = 0.015), 6 (p = 0.011) and 12 weeks (p = 0.003) with greater lightening in the gum-group at each examination period.

**Conclusion:**

These results support the efficacy of the tested nicotine replacement gum in stain reduction and shade lightening. These findings may help dentists to motivate those wishing to quit smoking using a nicotine replacement gum.

**Trial registration:**

NCT01440985

## Background

Development of nicotine replacement products with oral benefits could provide smokers with additional motivation to quit smoking and give dental professionals an additional reason to introduce the topic of smoking cessation with their patients. Availability of a smoking cessation product with stain removal or tooth whitening activity would provide an opportunity for dentists to illustrate some early measurable benefits of smoking cessation, reinforce smokers’ commitment to quit and support them on the path to a stable state of not smoking.

Nicotine replacement therapy (NRT) is the most widely-used effective treatment for tobacco dependence [1]. One of the most common cosmetic effects of cigarette smoking is the deposition of heavy stain on teeth. Results from in vitro tests suggest that Nicorette® Freshmint Gum (nicotine polacrilex gum) used for smoking cessation may have stain reducing (whitening) effects on smokers’ teeth [2]).

The act of gum chewing is associated with several beneficial effects including increased saliva output and mechanical removal of debris and dental stains [3]. Nicorette® Freshmint Gum also contains NaHCO3 (sodium bicarbonate), Na2CO3 (sodium carbonate) and CaCO3 (calcium carbonate) - three ingredients also found in commercially available tooth-whitening products; and xylitol, a chelating agent non-fermentable sugar which stimulates salivary flow and interferes with bacterial adhesion to oral tissues thus helping to reduce the risk of caries [4,5]. The combination of high-pH buffers (sodium bicarbonate, sodium carbonate) and an abrasive agent (calcium carbonate) in conjunction with salivary stimulation resulting from the gum flavour (xylitol) and chewing action are likely to have a positive impact on stained teeth while the gum is being used for smoking cessation.

This hypothesis is supported by data from a recent in vitro trial which demonstrated that the nicotine replacement gum (2 mg and 4 mg strengths) was comparable with, or better than, some leading teeth-whitening brands of confectionary gums [2].

According to the literature, other studies performed with commercial whitening gums demonstrated a reduction in the stain index from week 2 onwards [6-13]. Subjects in these studies may have been partially supervised and, most importantly, the trial designs did not specifically address smokers.

The hypothesis tested in this study was that a nicotine replacement gum removes more stain and whitens teeth more during a 6-week smoking cessation programme than a nicotine replacement sub-lingual tablet.

## Methods

The objectives of this study were to assess extrinsic stain reduction from baseline while quitting smoking using either Nicorette® Freshmint Gum (nicotine polacrilex) or Nicorette® Microtab (nicotine beta-cyclodextrin); a neutral sublingual tablet with no whitening properties compared to the gum. The primary efficacy parameter was the mean change in the modified extrinsic tooth stain score (all sites) between baseline and 6 weeks as this was consistent with the duration of other whitening studies. A secondary outcome measures was the change in tooth shade, measured by the Vita Shade Guide, between baseline and Weeks 2, 6 and 12.

The duration of the current trial in smokers was 12 weeks, with observations at weeks 2, 6 and 12.

Thus, the study was an evaluator-blinded, randomized, 12-week parallel group controlled trial which compared the stain reduction efficacy of a nicotine replacement gum against the reference product a nicotine replacement sub-lingual tablet in healthy smokers motivated to quit smoking and with visible staining of teeth. It was conducted in compliance with the ethical principles originating in or derived from the Declaration of Helsinki and with all International Conference on Harmonization (ICH) Good Clinical Practice (GCP) Guidelines [14]. The Irish Medicines Board and the Clinical Research Ethics Committee of The Cork Teaching Hospitals, approved the study, trial participants provided informed consent.

Stain was measured using the MacPherson Modification of the Lobene Stain Index [9] which scores eight sites per tooth, four facial and four lingual or palatal. The stain score per tooth site was determined by multiplying the score for stain intensity (scores 0-3) by that for stain area (scores 0-3). The test teeth were the eight incisors; if one of the eight incisors was not present or scorable, a canine was substituted. The stain score per subject was determined by averaging scores across tooth sites for that subject.

The intrinsic tooth shade was visually graded using a traditional Vita® Lumin Shade guide as a reference standard (VITA Zahnfabrik, H.Rauter GmbH & Co. KG, D-79713 Bad Säckingen, Germany). The shade assessments were made under standardized lighting conditions: assessments were all conducted in the same windowless room using color-corrected lighting in the range of 5000 degrees Kelvin, with the subject seated in a special upright examining chair with the arch tooth position parallel to the floor. A blue bib was placed over the subject's clothing, and all lipstick was removed before scoring. Color shade values for the upper right central incisor and upper left lateral incisor were determined by selecting one Vita® Lumin Shade sample that most closely matched each tooth. For analysis, each of the 16 shade tabs was assigned a number from 1 (dark) to 16 (light) according to the Munsell colour ranking system [15] as follows:

C4 = 1 A4 = 2 C3 = 3 B4 = 4

A3.5 = 5 B3 = 6 D3 = 7 A3 = 8 D4 = 9

C2 = 10 C1 = 11 A2 = 12 D2 = 13

B2 = 14 A1 = 15 B1 = 16

An increase in difference from baseline after treatment suggests an improvement in tooth shade. Subject scores were computed for each visit by averaging individual scores across teeth. Change from baseline was computed for each subject using these averages.

Inclusion criteria were a minimum of 20 natural teeth present with at least 10 of the 12 anterior teeth present and scorable, and a total extrinsic facial tooth stain score ≥ 28 according to Lobene stain index. Teeth that were grossly carious, fully crowned, or extensively restored on the facial or lingual surfaces were not included in the tooth count.

**Figure 1:**
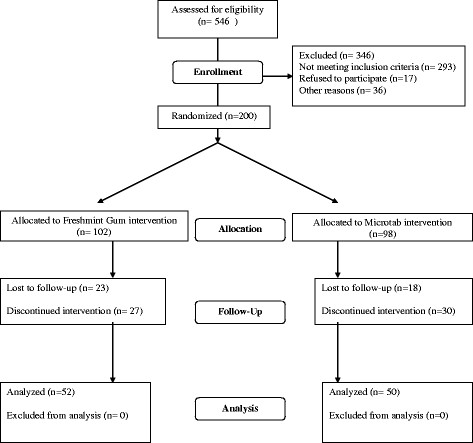
**Flow of subjects through tooth stain removal study.** The flow of subjects through the study is shown according to the CONSORT Statement. [16]

The trial was carried out at the Oral Health Services Research Centre in University College Cork over a 5 month period between July and November. The flow of subjects into and through the trial is illustrated in the CONSORT flow chart [16] in Figure 1. Of the 546 adults assessed for eligibility, 200 smokers were randomized at baseline to receive either the nicotine replacement gum or the nicotine replacement tablet to help them quit smoking. Reasons for non-enrollment are outlined in Figure 1. Subjects were classified according to the Fagerström Test for Nicotine Dependence (FTND) [17]. The randomization schedule was produced by the sponsor using an SAS Based Randomization Generator. The randomization was stratified according to 8 combinations of nicotine dependence level (defined as Low = Fagerström Total ≤ 5 and High = Fagerström Total ≥ 6) and baseline facial stain level (defined as sum of the facial scores equal to 28-49, 50-74, 75-99, or ≥ 100). Participants were allocated to groups at the study site by the local clinical trial coordinator, according to the randomisation schedule. The examiner was blind to the group allocation and participants were asked not to break the examiner blinding. High-nicotine dependent smokers (FTND ≥ 6,) received nicotine 4 mg gum or were instructed to use a 4 mg dosage of the tablet (2 tablets); low-nicotine dependent smokers (FTND ≤ 5) received nicotine 2 mg gum or were instructed to use a 2 mg dosage of the tablet (one tablet). The trial comprised of five visits: Baseline (entry visit), week one, week 2, week 6 and week 12 (study end). A trained and calibrated examiner rated tooth stain and shade at baseline and weeks 2, 6 and 12. Intra-examiner reliability was checked by repeat examination of 11 subjects with dental staining prior to study examinations. At all visits after baseline, smoking status and use of study treatment (gum or tablets) was checked. All subjects were instructed to use the chewing-gum or sublingual tablet for 12 weeks and to quit smoking the day after enrolment. Subjects were advised to use the trial medication (gum or tablet) frequently in accordance with product labelling to minimize symptoms of tobacco deprivation. The maximum recommended dosage per day was 15 x 4 mg pieces of gum or 40 x 2 mg tablets for the high-nicotine dependent group, and 15 x 2 mg pieces of gum or 20 x 2 mg tablets for the low-nicotine dependent group. Smoking status was assessed by self-reported abstinence and biochemically verified by measuring the level of CO in exhaled air, using a Bedfont monitor [18].

Oral care was standardized throughout the trial. At baseline, subjects were given a soft-bristled toothbrush and regular fluoride toothpaste and instructed to brush their teeth no more than twice a day. Use of any other oral hygiene or tooth-whitening product was prohibited during the trial. There were no dietary or drink restrictions. All observed or volunteered Adverse Events, the severity (mild, moderate, or severe) of each event, and the investigator’s opinion of the relationship to the trial medication were recorded.

## Statistical analysis

Using results from two previous oral care studies using the same index, a reasonable estimate of the standard deviation for the mean stain score was 1.1. Given this standard deviation, the current trial needed 50 completers per treatment group to have 90% power to detect a 0.73 unit difference. Assuming a 50% dropout rate, a sample size of 100 subjects per group at baseline (total of 200 subjects enrolled) was planned.

Analyses of primary and secondary variables were based on data from modified intent-to-treat (ITT) subjects, defined as all randomized subjects who had used the clinical trial test products and who had assessments at baseline and post-baseline. Abstinence from smoking was self-reported, and validated by an expired CO level of less than 10 ppm.

The primary outcome variable was the mean change in modified extrinsic tooth stain index (all sites) from baseline to week 6; this study duration was consistent with other tooth whitening studies in the literature [3,10-13] and was also consistent with the FDA´s definition of the primary time-point for smoking cessation efficacy (6 weeks with complete abstinence for the last four weeks).The full study ran for 12 weeks according to the design of traditional smoking cessation studies. The secondary outcome variables were: the mean change from baseline in modified extrinsic tooth stain score (based on all scored sites) at weeks 2 and 12 and in surface stain scores at different sites, stain area and intensity at weeks 2, 6 and 12; the change from baseline in tooth shade measured using the Vita® Shade Guide at weeks 2, 6, and 12; the number of gums or tablets used per day and throughout the trial period; and the number of cigarettes smoked per day.

Demographic and baseline characteristics were compared across treatment groups using either analysis of variance (ANOVA) or a chi-square test. If the expected number of subjects within a specific category was sufficiently small, Fisher’s exact test was used instead of the chi-square test. Treatments were compared using analysis of covariance (ANCOVA), using treatment and nicotine dependence as factors and the corresponding baseline measurement as a covariate. Each comparison was tested at the 0.05 level, two-sided.

## Results

Repeat examination of 11 subjects with dental staining resulted in ϰ statistics of 0.88 and 0.84 in intensity and area for intra-examiner reliability. A value of 0.99 was achieved for vita shade. Two hundred subjects (mean age 35.7 years) were enrolled in the study (Table 1). The flow of participants throughout the study is illustrated according to the CONSORT statement in Figure 1[16]. At baseline, subjects smoked a mean of 19.2 ± 8.0 cigarettes/day, their mean FTND score was 4.5 ± 2.44; 57% of subjects had made 2-5 quit attempts. At baseline, the total mean stain index was 4.2 ± 1.53 and the mean Vita® Shade score was 10.4 ± 3.24. One hundred and two subjects received nicotine gum, and 98 received nicotine tablets. A total of 102 subjects completed the 12-week trial.

**Table 1:** Summary of Demographic and Baseline Variables (All Randomized Subjects)

**Variables**	**Gum**		**Tablet**		**Total**	
	**(N = 102)**		**(N = 98)**		**(N = 200)**	
**Age (years)**						
Mean	35.2		36.3		35.7	
**Sex**						
Male	50	(49.0%)	54	(55.1%)	104	(52.0%)
Female	52	(51.0%)	44	(44.9%)	96	(48.0%)
**Cigarettes/Day**						
Mean	18.7		19.8		19.2	
Median	20.0		20.0		20.0	
Min,Max	(5,40)		(5,60)		(5,60)	
**Fagerström Test for Nicotine Dependence (FTND) Score Total**						
Mean	4.5		4.4		4.5	
Median	4.0		4.5		4.0	
Min,Max	(0,9)		(0,10)		(0,9)	
**Total Mean Stain Index**						
Mean	4.1		4.3		4.2	
S.D.	1.5		1.5		1.5	
Median	4.3		4.3		4.3	
**Vita**® **Shade Score**						
Mean	10.5		10.2		10.4	
S.D.	3.3		3.1		3.2	
Median	11.0		10.0		11.0	

## Smoking status

The intent-to-treat abstinence rates at week 6 were 41.2% (42/102) in the gum group and 36.7% (36/98) in the tablet group; at week 12, 35.3% (36/102) in the gum group and 37.8% (37/98) in the tablet group. The mean self-reported numbers of cigarettes smoked per day in the gum group and tablet group at weeks one, 2, 6 and 12 was less than or equal to 1.05 cigarette per day. The average use of gum or tablets was 8-9 pieces of 2 or 4mg gum and 8-16 tablets per day according to nicotine dependence category.

## Efficacy results

The primary outcome variable was the mean change in stain index scores between baseline and week 6. The level of stain was lower at week 6 than at baseline in the gum group (test) with a mean reduction of -0.14 and higher than at baseline in the tablet group (control) with a mean increase of 0.12 (Table 2). This difference in mean change in stain index scores between baseline and week 6 was statistically significant in favour of gum (p 0.005, ANCOVA).

At week 2 the mean change in stain index scores from baseline indicates a stain reduction in the gum group (-0.02) and an increase in the tablet group (0.05), however the difference in these mean changes were not significant (p = 0.147). At week 12, mean change in stain index scores in the gum and tablet group were -0.7 and -0.5 respectively, indicating stain reduction from baseline in both groups with no significant difference between the groups (p = 0.74).

Concerning site and area specific changes, statistically significant improvements in lingual stain index, body region stain index, and total stain area were noted with the gum group compared with the tablet group. At week 6, treatment with gum did not improve facial stain index or total stain intensity; however, the increases in facial stain index and total stain intensity in the gum group were smaller than the corresponding increases in the tablet group.

The change from baseline in the tooth shade at weeks 2, 6 and 12 were measured as secondary outcome variables. There was a greater degree of shade lightening in the gum group compared with the tablet group, p = 0.015, 0.011, 0.003 at 2, 6 and 12 weeks respectively (Table 3). Whilst the change in scores from baseline indicated a progressive lightening at the three time points in the gum group, the change in shade scores in the tablet group showed a darkening at weeks 2 and 6 and the mean change from baseline at week 12 was zero.

**Table 2:** Tooth –wise Mean Stain Index Total (Intent-To-Treat Subjects)

	**Week 2**		**Week 6**		**Week 12**	
	**Gum**	**Tablet**	**Gum**	**Tablet**	**Gum**	**Tablet**
**N**	78	70	60	59	52	50
**Baseline**						
Mean	4.1	4.3	4.2	4.3	4.2	4.4
S.D.	1.6	1.6	1.6	1.6	1.7	1.6
Median	4.3	4.2	4.3	4.3	4.4	4.4
Min,Max	(1.30,7.52)	(0.73,7.77)	(1.30,7.52)	(0.73,7.77)	(1.30,7.52)	(1.31,7.77)
**Post**						
Mean	4.1	4.3	4.0	4.4	4.1	4.4
S.D.	1.6	1.6	1.7	1.7	1.7	1.6
Median	4.1	4.3	4.2	4.6	4.4	4.1
Min,Max	(1.22,7.27)	(0.75,7.73)	(1.02,7.45)	(0.69,7.45)	(1.19,7.11)	(1.17,7.63)
**Change**						
Mean	-0.02	0.05	-0.14	0.12	-0.07	-0.05
S.D.	0.34	0.33	0.46	0.50	0.46	0.60
Median	-0.01	0.05	-0.10	0.09	-0.05	-0.02
Min,Max	(-0.83,0.73)	(-1.13,0.98)	(-1.41,0.97)	(-0.97,1.98)	(-1.41,0.88)	(-1.27,1.30)
Paired *t*-test p-value	0.536	0.191	0.018	0.079	0.251	0.588
LsMean	-0.03	0.05	-0.13	0.12	-0.09	-0.05
s.e.	0.04	0.04	0.06	0.06	0.08	0.078
**Comparison vs. Microtab**						
p-value	0.147^a^		0.005^a^		0.74^a^	
Difference	-0.08		-0.25		-0.04	
s.e.	0.06		0.09		0.11	
95% C.I.	[-0.19,0.03]		[-0.43,-0.08]		[-0.25,0.17]	

^a^P-values are based on ANCOVA model with terms for Treatment.

Note: Lower number indicates less stain.

**Table 3:** Vita® Shade Tooth Assessment (Intent-To-Treat Subjects)

	**Week 2**		**Week 6**		**Week 12**	
	**Gum**	**Tablet**	**Gum**	**Tablet**	**Gum**	**Tablet**
**N**	78	70	60	59	52	50
**Baseline**						
Mean	10.8	10.0	10.8	10.1	10.9	10.1
S.D.	3.2	3.2	3.15	3.23	3.15	3.18
Median	11.8	10.3	11.8	10.5	11.8	10.5
Min,Max	(2.0,15.0)	(3.0,15.0)	(2.5,15.0)	(3.0,15.0)	(2.5,15.0)	(3.0,15.0)
**Post**						
Mean	10.9	10.0	11.1	10.1	11.4	10.1
S.D.	3.1	3.2	3.1	3.2	3.2	3.2
Median	12.0	10.1	12.0	10.5	12.0	10.8
Min,Max	(2.0,15.0)	(3.0,15.0)	(2.0,15.0)	(3.0,15.0)	(2.0,15.0)	(2.0,15.0)
**Change**						
Mean	0.16	-0.03	0.28	-0.06	0.50	0.00
S.D.	0.70	0.17	0.94	0.55	1.16	0.64
Median	0.00	0.00	0.00	0.00	0.00	0.00
Min,Max	(-1.00,4.00)	(-1.00,0.50)	(-1.00,4.00)	(-2.00,1.00)	(-1.00,4.00)	(-2.00,2.00)
Paired *t*-test p-value	0.047	0.159	0.023	0.411	0.003	>0.999
LsMean	0.17	-0.04	0.25	-0.11	0.44	-0.12
s.e.	0.06	0.06	0.10	0.10	0.13	0.14
p-value	0.015^a^		0.011^a^		0.003^a^	
Difference	0.21		0.36		0.56	
s.e.	0.09		0.14		0.18	
95% C.I.	[0.04,0.38]		[0.09,0.64]		[0.19,0.92]	

^a^P-values are based on ANCOVA model with terms for Treatment.

Note: Based on Munsell color ranking from 1 = darkest to 16 = lightest shade.

The most common treatment-related adverse events were gastrointestinal disorders (reported by 21.6% of gum and 36.7% of tablet users), headache (22.5% gum vs. 17.3% tablet), sore mouth, hiccups, and cough. Most treatment-related adverse events were mild and transient. No serious treatment-related adverse events occurred during the study. One subject in the gum group discontinued treatment because of mild nausea and headache that were probably or possibly related to treatment.

## Discussion

The improvements in staining that occurred in the gum group were primarily due to reduced staining on the lingual surfaces. The reductions in the stain index (all surfaces) were primarily due to reductions in stain area, rather than reductions in intensity. These findings suggest that the gum had most impact on removal of newer stain, and less impact on removal of older stain. Although the stain index reduction from baseline in the gum group is modest, it is worth noting that, in contrast, the tablet group showed an increase in stain from baseline. This increase in stain in the tablet group is possibly a result of the dietary habits of this traditionally heavy tea-drinking population. Thus the reduction in stain in the gum group suggests an inhibitory effect on stain formation and progression by the gum in addition to the statistically significant modest stain removal found in this study.

The stain reduction in the gum group was not statistically significant at 12 weeks although the shade score was statistically significantly lighter (p = 0.003). The results of this study suggest that stain removal and tooth whitening are added benefits of using the nicotine replacement gum. These findings may offer an added incentive for smokers to quit smoking. The dental practitioner is well placed to encourage smokers to quit [19] and with the advantage of tooth whitening for their patients, may find it easier to broach the subject of smoking cessation. The results of this study are relevant to other health care workers who recommend nicotine replacement gum as the improvement in tooth stain and shade were not dependent on the dental setting because the study design did not incorporate any professional cleaning of the teeth.

## Conclusion

The results of this study confirm that chewing the tested nicotine replacement gum as recommended in a ‘real world’ active smoking cessation program produces a statistically significant change in the parameter of whitening as measured by change from baseline versus the negative control (Microtab) following 6 weeks in a smoking cessation programme. The Vita® Shade Guide (the secondary outcome measure) supported the trend of stain improvement. These results support the efficacy of the tested nicotine replacement gum in stain reduction, in arresting the progression of tooth stain and in shade lightening.

## Competing interests

One of the co-authors is Fredrik Nilsson who recently retired from his position as Senior Clinical Scientist with McNeil AB, the company who manufacture the test and control products. The other authors have no competing interests.

## Authors’ contributions

HW was the principal investigator of the project, participated in the design of the study, applied for ethical approval, coordinated the study, input into the data analysis, interpreted the results and drafted the manuscript. RK carried out the clinical examinations. DOM participated in the design of the study, input into the analysis and interpretation of results and commented on the manuscript. FN led the design of the study, trained the team in the smoking cessation intervention and monitoring, and oversaw the statistical analyses. All authors read and approved the final manuscript.

## Pre-publication history

The pre-publication history for this paper can be accessed here:

http://www.biomedcentral.com/1472-6831/12/13/prepub
